# Steroid receptor coactivator 1 promotes human hepatocellular carcinoma invasiveness through enhancing MMP‐9

**DOI:** 10.1111/jcmm.18171

**Published:** 2024-03-20

**Authors:** Zhangwei Tong, Yong Zhang, Peng Guo, Wei Wang, Qiang Chen, Jing Jin, Shixiao Liu, Chundong Yu, Pingli Mo, Lei Zhang, Junli Huang

**Affiliations:** ^1^ State Key Laboratory of Cellular Stress Biology, Innovation Center for Cell Biology, School of Life Sciences Xiamen University Xiamen China; ^2^ Department of Molecular and Cellular Biology Baylor College of Medicine Houston Texas USA; ^3^ Department of Cardiology, School of Medicine The First Affiliated Hospital of Xiamen University, Xiamen University Xiamen China; ^4^ Hepatic Surgery Center, Tongji Hospital, Tongji Medical College Huazhong University of Science and Technology Wuhan China; ^5^ Department of Hepatobiliary Surgery, Shanxi Bethune Hospital, Shanxi Academy of Medical Sciences Shanxi Medical University; Shanxi Tongji Hospital, Huazhong University of Science and Technology Taiyuan China; ^6^ Department of General Surgery Army 73rd Group Military Hospital of the Chinese People's Liberation Army (Chenggong Hospital of Xiamen University) Xiamen China

**Keywords:** cell invasiveness, hepatocellular carcinoma (HCC), MMP‐9, steroid receptor coactivator 1 (SRC‐1), transcriptional activity

## Abstract

SRC‐1 functions as a transcriptional coactivator for steroid receptors and various transcriptional factors. Notably, SRC‐1 has been implicated in oncogenic roles in multiple cancers, including breast cancer and prostate cancer. Previous investigations from our laboratory have established the high expression of SRC‐1 in human HCC specimens, where it accelerates HCC progression by enhancing Wnt/beta‐catenin signalling. In this study, we uncover a previously unknown role of SRC‐1 in HCC metastasis. Our findings reveal that SRC‐1 promotes HCC metastasis through the augmentation of MMP‐9 expression. The knockdown of SRC‐1 effectively mitigated HCC cell metastasis both in vitro and in vivo by suppressing MMP‐9 expression. Furthermore, we observed a positive correlation between SRC‐1 mRNA levels and MMP‐9 mRNA levels in limited and larger cohorts of HCC specimens from GEO database. Mechanistically, SRC‐1 operates as a coactivator for NF‐κB and AP‐1, enhancing MMP‐9 promoter activity in HCC cells. Higher levels of SRC‐1 and MMP‐9 expression are associated with worse overall survival in HCC patients. Treatment with Bufalin, known to inhibit SRC‐1 expression, significantly decreased MMP‐9 expression and inhibited HCC metastasis in both in vitro and in vivo settings. Our results demonstrated the pivotal role of SRC‐1 as a critical modulator in HCC metastasis, presenting a potential therapeutic target for HCC intervention.

## 1 INTRODUCTION

With approximately 841,000 new cases and 782,000 fatalities annually, liver cancer stands as the sixth most commonly diagnosed cancer and the fourth leading cause of cancer‐related deaths worldwide. Furthermore, it holds the position of being the second most prevalent cancer in men, surpassed only by lung cancer.^1^ Primary liver cancer is mainly composed of hepatocellular carcinoma (HCC) (75%–85% of cases) and intrahepatic cholangiocarcinoma (10%–15% of cases), in addition to other rare types.^1^ HCC is characterized by a challenging clinical scenario marked by a poor prognosis, rapid growth, vascular invasion, metastasis and frequent recurrence. Given the elevated incidence and mortality rates associated with HCC, unravelling the molecular mechanisms driving its metastasis becomes imperative. Despite considerable efforts, the scientific community still lacks a comprehensive understanding of the intricate molecular processes underpinning HCC metastasis. The absence of effective medications to prevent metastasis further accentuates the urgency of advancing our comprehension in this critical area.

As an actual nuclear receptor (NR) coactivator, the steroid receptor coactivator 1 (SRC‐1, also known as NCOA1) was originally identified.^2^ It is a member of the SRC/p160 family along with SRC‐2 (also known as NCOA2) and SRC‐3 (also known as AIB1 and NCOA3).^3^, ^4^ SRC‐1 acts as a transcriptional coactivator for steroid receptors, including the oestrogen receptor (ER)^5^ and androgen receptor (AR),^6^ and other transcriptional factors, containing PEA3,^7^ Ets‐2,^8^ NF‐κB^9^ and AP‐1.^10^

SRC‐1 is shown to play oncogenic roles in several cancers, containing breast cancer and prostate cancer. SRC‐1 expression is associated with HER2 positivity, overall survival, disease recurrence and resistance to endocrine therapy.^11^, ^12^ Disruption of the SRC‐1 gene in mice inhibited breast cancer metastases without impeding the formation of primary tumours.^13^ SRC‐1 promotes invasive and metastatic breast cancer by co‐activating PEA3 mediated Twist expression.^7^ The expression of SRC‐1 is associated with clinical and pathological variables indicative of heightened tumour aggressiveness in clinically localized, androgen‐dependent cancers. Noticeably reduced SRC‐1 expression is linked to diminished growth and alterations in AR target gene regulation in LNCaP and C4‐2 cell lines.^14^ Our lab previously reported that SRC‐1 is highly expressed in 25 of 40 human HCC specimens. Down‐regulation of SRC‐1 decreased HCC cell proliferation and impaired tumour maintenance in HCC xenografts, and knockdown of SRC‐1 reduced protein levels of the proliferation marker proliferating cell nuclear antigen (PCNA) and the oncogene c‐Myc. And knockout of SRC‐1 in mice reduced diethylnitrosamine/CCl4‐induced tumour formation in the liver and the expression of c‐Myc and PCNA in liver tumours. Mechanically, SRC‐1 promotes HCC progression by enhancing Wnt/β‐catenin signalling.SRC‐1 promotes HCC progression by enhancing Wnt/beta‐Catenin signalling.^15^ Whereas, the character of SRC‐1 in HCC metastasis maintains undiscovered.

Matrix metalloproteinase 9 (MMP‐9), also known as gelatinase B, which pertains to the MMP family of 24 zinc‐dependent endopeptidases that is referred to the ruin of extracellular matrix.^16^ MMP‐9 previously was identified to act crucial character in tumour invasion, metastasis and angiogenesis. And it also can regulate tumour microenvironment, which is a vital biomarker for a variety of cancers.^16^ MMP mainly displays its function in gene transcription, protein translation, pro‐MMP activation, and endogenous inhibition. SRC‐1 directly binds PEA3 and binds to VEGF and MMP‐9 promoter in glioma cell.^17^ The interaction of SRC‐1 and Ets2 regulates MMP9 target genes expression in Aromatase inhibitors resistance in breast cancer cells.^18^ However, whether SRC‐1 could regulate MMP9 expression in HCC cells and the molecular mechanism are still unknown.

NF‐κB is a protein multimers that regulates gene transcription, cytokine production and cell survival.^19^ NF‐κB acts a widely known role in modulation of immune responses and inflammation, while increased evidences sustain crucial function in oncogenesis.^20^ NF‐κB activation due to various signalling pathways caused by several cytokines, growth factors and tyrosine kinases.^20^ Upon activation, NF‐κB is translocated to cell nucleus and then performs its function as transcription factor. Activator protein 1 (AP‐1) is a crucial transcription factor which modulates target gene expression upon multiple stimulation, including cytokines, bacterial and viral infections.^21^, ^22^ AP‐1 orchestrates various cellular processes, encompassing differentiation, proliferation and apoptosis. Structurally, AP‐1 forms a heterodimer comprising proteins from the c‐Fos, c‐Jun, ATF and JDP families. Its functions predominantly hinge on the specific Fos and Jun subunits, which collectively contribute to the formation of AP‐1 dimers.^21^, ^22^ SRC‐1 couples on NF‐κB (p50/p65), and the coactivation complex first hand facilitated VEGF transcription in thyroid cancer.^23^ SRC‐1 enhances ITGA5 promoter activity via an AP‐1 binding site in breast cancer cells to promote breast cancer metastasis.^10^ In HCC cells, the question of whether SRC‐1 coactivates NF‐κB or AP‐1 has remained elusive. This study discloses SRC‐1 as a promoter of HCC metastasis by orchestrating the upregulation of MMP9. SRC‐1‐knockdown demonstrated a substantial reduction in HCC cell metastasis, both in vitro and in vivo, attributed to the inhibition of MMP‐9 expression. Positive correlations were observed between SRC‐1 mRNA levels and MMP‐9 mRNA levels in both a limited cohort of HCC specimens and a larger cohort obtained from the GEO database. SRC‐1 functions as coactivators for both NF‐κB and AP‐1, thereby regulating MMP‐9 promoter activity in HCC cells. Elevated levels of SRC‐1 and MMP‐9 expression are associated with a worse overall survival in HCC patients. Treatment with Bufalin, known for its ability to inhibit SRC‐1 expression, significantly reduced MMP‐9 expression and effectively inhibited HCC metastasis both in vitro and in vivo. In conclusion, our findings underscore SRC‐1 as a pivotal regulator in HCC metastasis and propose it as a potential therapeutic target for HCC therapy.

## 2 MATERIALS AND METHODS

### 2.1 Cell culture and chemical treatment

HepG2 and MHCC97H human HCC cells obtained from Cell Bank of Type Culture Collection of Chinese Academy of Sciences (Shanghai, China) and preserved in our laboratory. The cell lines were cultured in DMEM medium with 10% foetal bovine serum and 100 U/mL penicillin–streptomycin. For 12‐O‐tetradecanoylphorbol‐13‐Acetate (TPA) treatment, cells were cultured in serum free medium with 100 nM TPA for 24 h. For Bufalin treatment, cells were cultured in DMEM medium with Bufalin of indicated concentration for 24 h.

### 2.2 Gene knockdown and Stable knockdown cell lines

Specific and non‐specific siRNAs were obtained from Invitrogen. siRNA was transfected by lipofectamine 2000 (Invitrogen) according to the manufacturer's instructions. To gain stably transfected cells, cells were transfected with the specific shRNA plasmids. stably knockdown cells were screened via puromycin. SRC‐1‐specific targeting sequences are CCTCAGGGCAGAGAACCATCT and CACGACGAAATAGCCATAC. To generate stably MMP‐9 rescued cells, SRC‐1‐knockdown cells were transfected with PCR3.1‐MMP‐9 plasmid and stably transfected cells were screened via G418.

### 2.3 Western blot

Cells were lysed in RIPA buffer containing proteinase inhibitors. Protein concentration was detected via BCA assay. Same amount of proteins in each cell lysate was separated on SDS‐PAGE and then transferred to PVDF membranes. Membranes were incubated with the following antibodies: SRC‐1 (cell signalling Technology), MMP‐9 (Abclonal), β‐actin (Sigma‐Aldrich). Membranes then were incubated with a horseradish peroxidase‐conjugated secondary antibody (Pierce). Immunoreactivities were visualized by using the ECL kit.

### 2.4 Transwell assay

Matrigel‐coated Millicell inserts (Millipore) were used for Matrigel invasion assays. 2.5 × 10^4^ cells were implanted per upper chambers in serum‐free medium, while the lower chambers were loaded with 10% FBS‐included medium. After 24 h, non‐migrating cells in the upper chambers were move away using a swab and cells infiltrating into the submembrane through the stromal layer were dyed and counted. Cell migration assays were also performed without Matrigel.

### 2.5 Real time PCR

Total RNA was extracted via Trizol reagent (Invitrogen) and reversed to cDNA using the Reverse Transcriptase Core Kit (Toyobo). Real‐time PCR were executed using SYBR Green Master (Roche). Relative quantification was normalized to GAPDH. Primers for SRC‐1 were 5’‐GTTCATCCGACCCTGCTAAC and 5’‐TCAAAATCTTGCATTTGTCTGG. Primers for MMP‐9 were 5’‐TCGTGGTTCCAACTCGGTTT and 5’‐GCGGCCCTCGAAGATGA. Primers for GAPDH were 5′‐AACTTTGGCATTGTGGAAGG and 5’‐GGATGCAGGGATGATGATGTTCT.

### 2.6 Zymography

Cells were inoculated in 6‐well plate and cultured with TPA (100 nM) for 24 h in serum‐free medium. Conditional medium was gathered and devoted to electrophoresis on 10% SDS–PAGE containing gelatin (1 mg/mL). The gels were washed using buffer I (50 mM Tris–HCl, pH 7.5 and 2.5% Triton X‐100) for 30 min, and then incubated in buffer II (150 mM NaCl, 5 mM CaCl2, 50 mM Tris–HCl, pH 7.6, 0.02% NaN3) for 48 h. The gels were then dyed with 0.25% Coomassie brilliant blue and photographed.

### 2.7 Human HCC specimens

The mRNA levels of SRC‐1 and MMP‐9 in HCC tissues were analysed, and the HCC tissues were obtained from the First Affiliated Hospital of Xiamen University (Xiamen, China). Informed consent was obtained from each patient and the research protocol conformed to the ethical guidelines of the 1975 Declaration of Helsinki was approved by the Institute Research Ethics Committee at Xiamen University.

### 2.8 Promoter/reporter assay

Promoter/reporter plasmids were transfected to cells, and PCR3.1‐Rluc plasmids were transfected together as internal control. Cells were harvested at 24 h or 48 h after transfection, with or without 100 nM TPA treatment. Luciferase activity was detected and normalized to the value of Renilla luciferase activity.

### 2.9 ChIP assay

Control and SRC‐1‐knockdown of MHCC97H cells were treated with TAP for 24 h, and then the chromatin was immunoprecipitated using anti‐c‐Jun, anti‐p65 or nonspecific IgG (Abcam, US) antibody. ChIP DNAs were purified and amplified by qRT‐PCR with specific primers for the MMP‐9 promoter. The MMP‐9 promoter primers for anti‐AP‐1 were as follow: F,5′‐GAGAGAGGAGGAGGTGGTGTAAG‐3′; R‐5′‐TGAGGGCAGAGGTGTCTGACT‐3′, the MMP‐9 promoter primers for anti‐p65 were as follow: F, 5′‐AAGAGAGTAAAGCCATGTCTGCTGT‐3′; R‐5’ GCTCTGTCCTCTTTTTCCCTCCC‐3′.

### 2.10 Nude mouse metastasis model

To evaluate tumour metastasis, a total of 5 × 10^6^ HepG2 cells were injected into nude mice via the tail vein. The mice were treated with bufalin (1 mg/kg) via an intraperitoneal injection for 3 weeks. After injection for 3 weeks, the mice were sacrificed, and the lungs were fixed for haematoxylin and eosin staining, and tumours in the lungs were quantitative.

### 2.11 Statistical analysis

Data were obtained from independent experiments, with three replicates performed in each experiment. All data are expressed as the mean ± S.E or mean ± SEM. Statistical differences were determined by two‐tailed Student's *t*‐test or One‐Way ANOVA, with *p* < 0.05 being considered significant.

## 3 RESULTS

### 3.1 Downregulation of SRC‐1 decreased cell invasion in HCC cells

**FIGURE 1:**
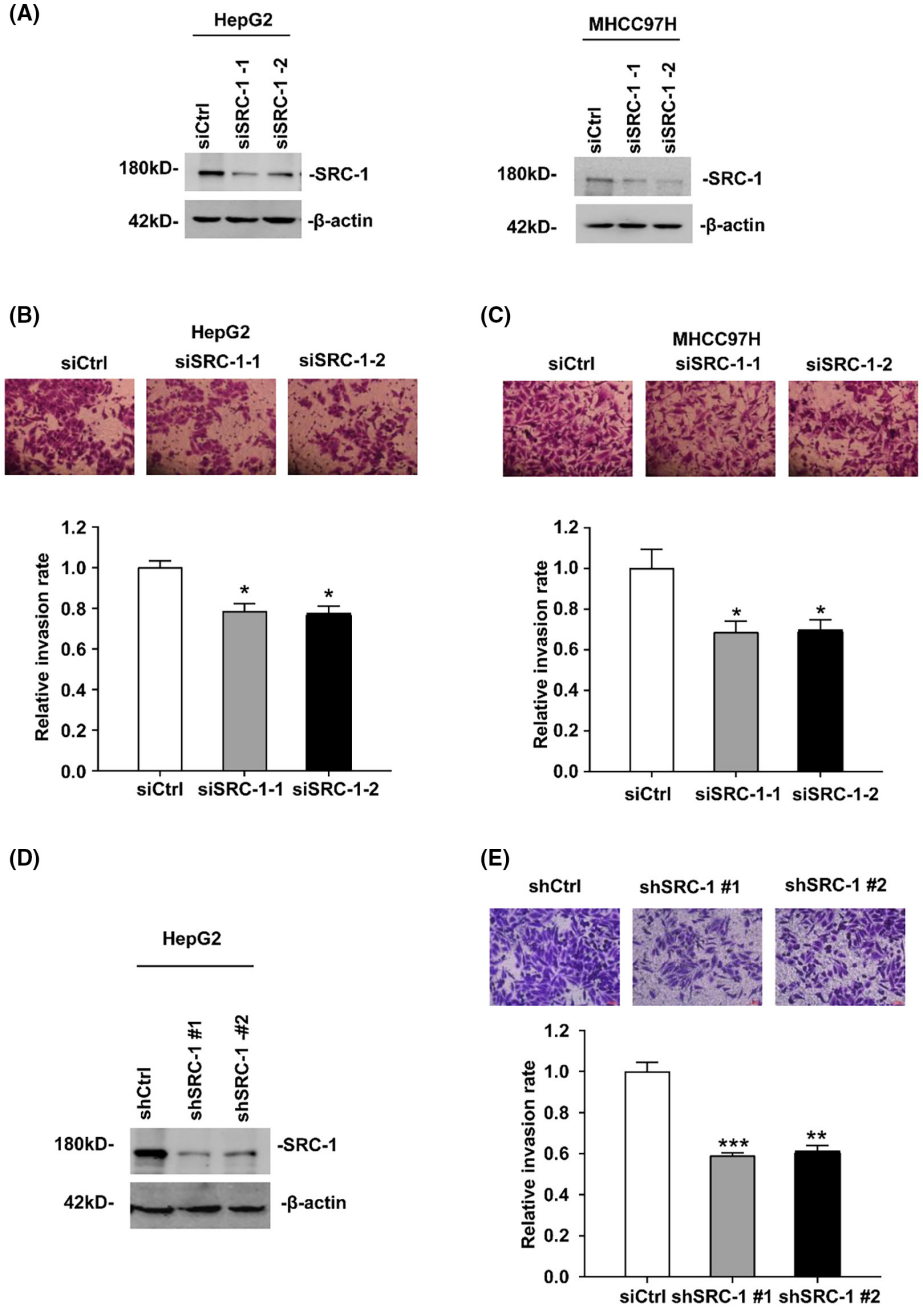
Downregulation of SRC‐1 decreases HCC cells invasion. (A) Western blot of transient knockdown of SRC‐1 in HepG2 and MHCC97H cells. (B, C) Images and quantitation of SRC‐1 transient knockdown and control cells infiltrate. All data are the means + sd. (D) the knockdown efficiency of SRC‐1 was measured in HepG2 by Western Blot. (E) Images and quantitation of SRC‐1 stable knockdown HepG2 cells penetrating through the matrigel‐coated membrane. *p < 0.05, **p < 0.01, ***p < 0.001.

We previously reported that SRC‐1 is higher expression in HCC cells, and facilitates HCC development via activating Wnt/β‐catenin signalling.^15^ However, it is unclear whether SRC‐1 possesses function in cell metastasis in HCC. To evaluate the function of SRC‐1 on cell invasiveness, we established SRC‐1‐knockdown in two HCC cell lines (HepG2 and MHCC97H) (Figure 1A). Cell invasiveness was measured by Matrigel embedded trans‐well assay. Comparing with cells that transfected with siCtrl RNA, the cell numbers that invaded through the Matrigel in SRC‐1 transiently knockdown groups were decreased nearly 30% (Figure 1B, C), suggesting that SRC‐1 may involve in cell invasiveness.

Then we stably knocked down SRC‐1 protein levels in HepG2 cells, as shown in Figure 1D. Consistent with the result that SRC‐1 transiently knockdown decreased cell invasiveness, SRC‐1 stably knockdown cells showed a decreased invasion by nearly 40%, in comparison with control cells (Figure 1E). To test whether SRC‐1 could affect cell migration in HCC cells, cell migration capacity was detected via transwell assay without Matrigel embedded. As shown in Figure S1A, SRC‐1 stable knockdown showed a lower migration rate than control cells, suggesting that SRC‐1 downregulation decreased cell migration in HCC cells. These results indicated that SRC‐1 can promote HCC cell metastasis.

### 3.2 Downregulation of SRC‐1 decreased MMP‐9 expression

**FIGURE 2:**
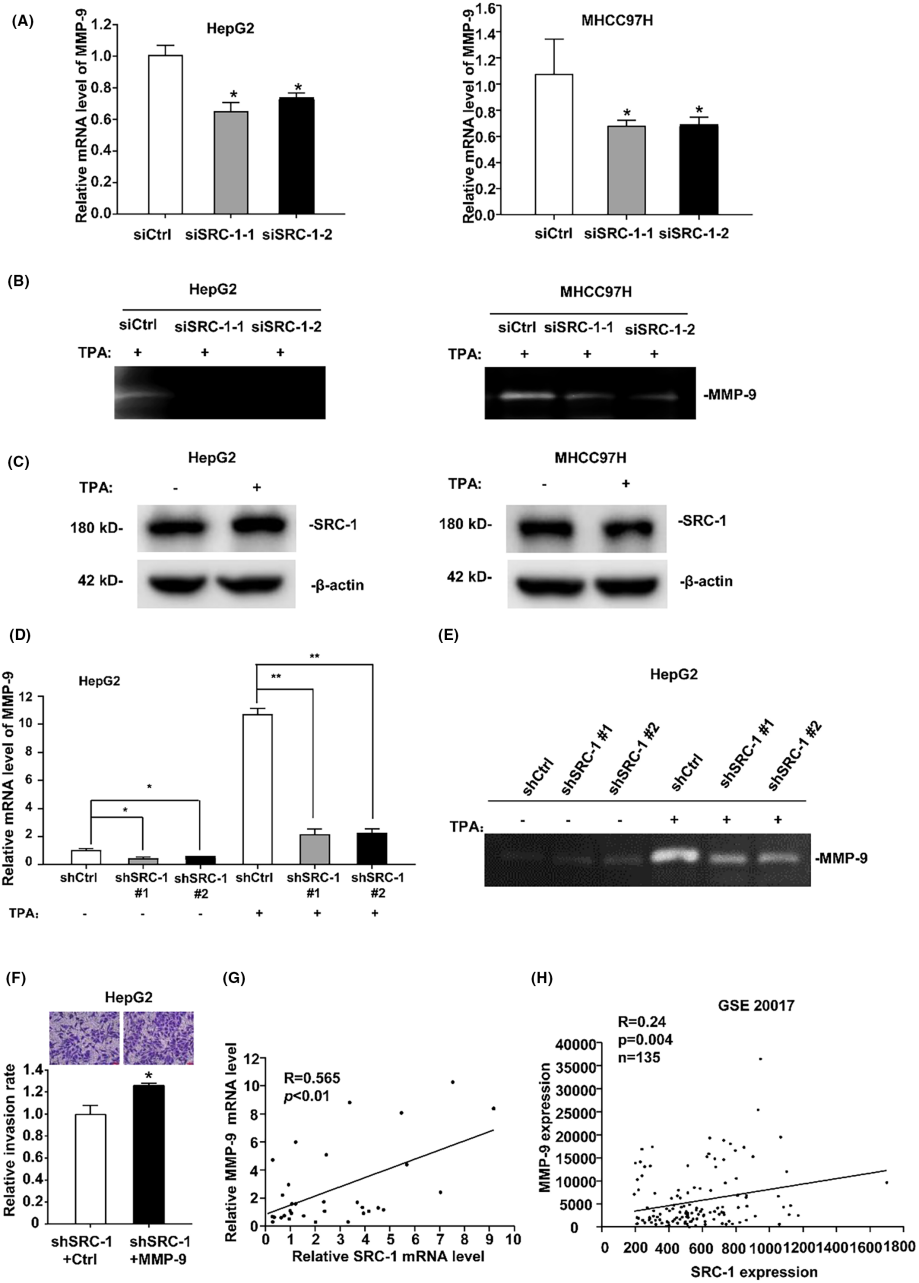
Downregulation of SRC‐1 decreased the expression of MMP‐9. (A) Transient knockdown of SRC‐1 decreases the MMP‐9 mRNA expression in HepG2 and MHCC97H cells. (B) MMP‐9 enzymatic activity was significantly decreased in SRC‐1 transiently knockdown cells when treated with TPA. (C) TPA‐induced SRC‐1 expression in HCC cells. (D, E) TPA‐induced MMP‐9 mRNA expression and enzymatic activity were obviously reduced compared with that in ctrl cells in SRC‐1 stably knockdown HepG2 cell lines. (F) SRC‐1 knockdown HepG2 cells transfected with MMP‐9 showed increased invasive ability. The calculated number from triplicate was shows Images and quantitation cells penetrating through the matrigel‐coated membrane. (G) The mRNA levels of SRC‐1 were positively related to MMP‐9 in human HCC patient specimens. (H) In GEO database GSE20017, the mRNA levels of SRC‐1 and MMP‐9 showed the positive correlation. *p < 0.05, **p < 0.01.

Given that SRC‐1 potentially facilitates HCC cell metastasis and invasion, our curiosity led us to investigate the underlying mechanism. Notably, our laboratory has previously reported that SRC‐3, another member of the SRC family, can elevate MMP‐9 activity, thereby enhancing HCC cell migration and invasion.^24^, ^25^ We sought to investigate whether SRC‐1 promotes HCC metastasis by enhancing MMP‐9. To assess this, we quantified the mRNA levels of MMP‐9 in SRC‐1 transient knockdown cells and control cells. As shown in Figure 2A, transient knockdown of SRC‐1 significantly inhibited MMP‐9 mRNA levels by nearly 40% in HepG2 and MHCC97H cells. Consistently, MMP‐9 enzymatic activity, which was measured by zymography assay, was obviously reduced in SRC‐1 transiently knockdown cells when treated with TPA (Figure 2B), which can increase the expressions of MMP‐9 via NF‐κB and AP‐1 signalling.^26^, ^27^ To investigate whether TPA affects the expression of SRC‐1, we initially examined the protein levels of SRC‐1 in HCC cells after TPA treatment. Western blot results demonstrated that TPA treatment did not significantly alter the protein levels of SRC‐1 (Figure 2C). These results showed that SRC‐1 regulated MMP‐9 mRNA levels and enzymatic activity. MMP‐2, another important MMP with a molecular weight of 72 kD, was not expressed in these HCC cells (HepG2 and MHCC97H), as no 72 kD band could be seen from the zymography gel treated with or without TPA treatment (data not shown), suggesting that MMP‐9 may be the main functional MMP in these HCC cells. In HepG2 cell lines with stable knockdown of SRC‐1, both MMP‐9 mRNA levels and enzymatic activity exhibited a significant decrease when compared with control cells (Figure 2D,E), in the presence or absence of TPA, confirming that SRC‐1 can regulate MMP‐9 expression. Further, we detected the effect of SRC‐1 overexpression on MMP‐9 expression in HCC cells. Western blot results showed that SRC‐1 overexpression significantly increased MMP9 protein levels in HCC cells (Figure S1B).

To confirm that decreased invasion in SRC‐1 knockdown cells were due to the loss of MMP‐9, we restored the MMP‐9 expression in SRC‐1‐knockdown‐ HepG2 cells. As shown in Figure S1C, MMP‐9 mRNA levels were significantly increased after stably transfected MMP‐9 in SRC‐1‐knockdown‐ HepG2 cells. Similarly, MMP‐9 enzymatic activity was significantly increased after stably transfected MMP‐9 in SRC‐1 knockdown HepG2 cells (Figure S1D). Restoration of MMP‐9 in SRC‐1 knockdown HepG2 cells could rescued the invasiveness and migration rate (Figure 2F and Figure S1E), indicating that SRC‐1 increased HCC metastasis through enhancing MMP‐9 expression. These results indicated that SRC‐1 increased cell metastasis through inducing MMP‐9 expression.

Our laboratory had previously gathered a collection of human HCC patient specimens, so we assessed the mRNA levels of both SRC‐1 and MMP‐9 within the same set of specimens. As showed in Figure 2G, a positive correlation was observed among human HCC specimens. Furthermore, in the GEO database GSE20017, encompassing a larger cohort of HCC specimens, the positive correlation between SRC‐1 and MMP‐9 was consistently validated. (Figure 2H). These results indicated that SRC‐1 may regulate MMP‐9 expression in HCC cell lines and human HCC specimens.

### 3.3 SRC‐1 regulated MMP‐9 promoter activity through coactivating NF‐κB and AP‐1

**FIGURE 3:**
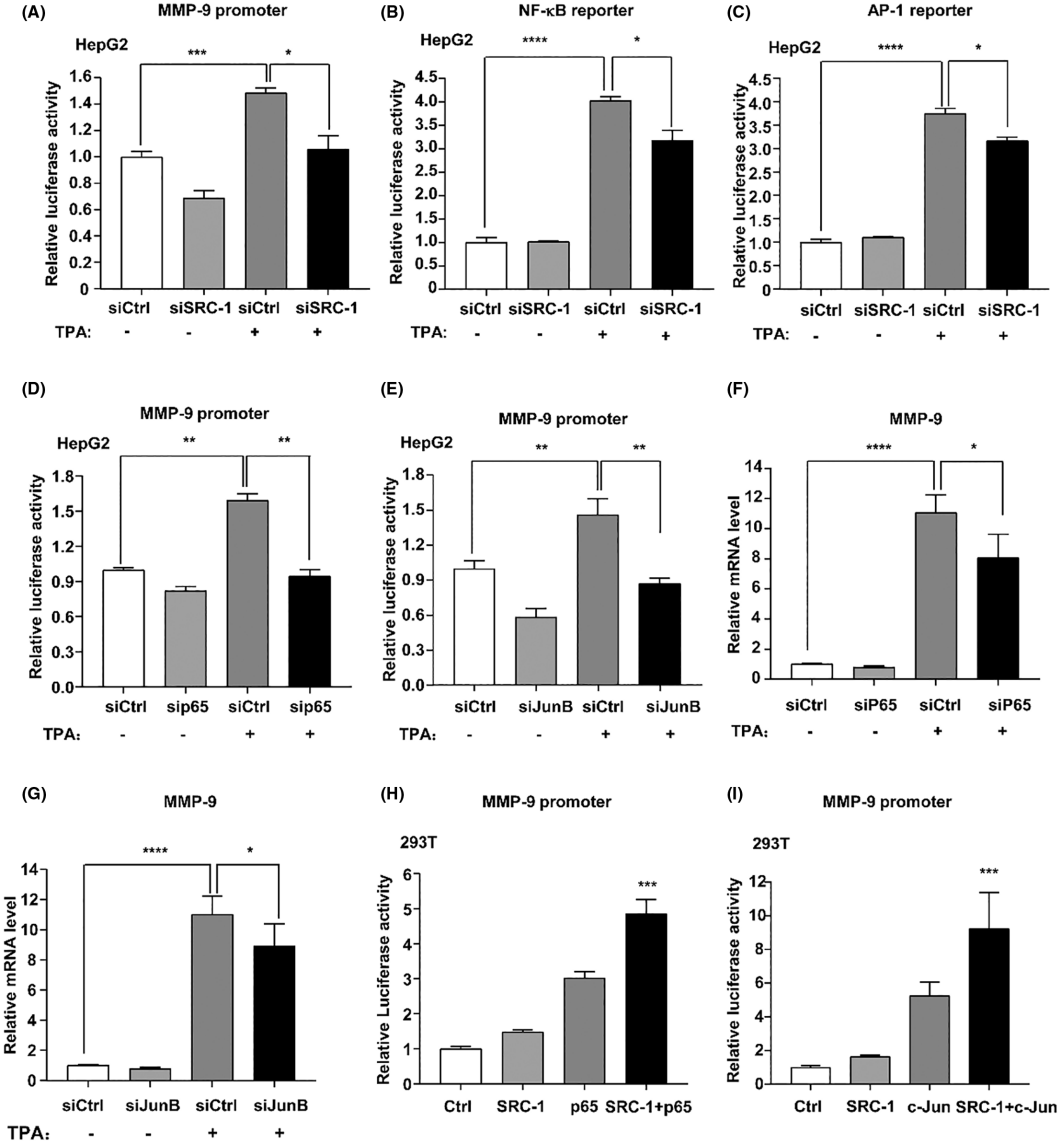
Knockdown of SRC‐1 reduced MMP‐9 promoter activity. (A–C) TPA‐induced MMP‐9, NF‐κB and AP‐1 transcriptional activity was observably reduced in SRC‐1‐knockdown cells. (D, E) TPA‐induced MMP‐9 promoter was observably reduced in P65‐knockdown cells and JunB‐knockdown cells. (F, G) TPA‐induced MMP‐9 mRNA was observably reduced in P65‐knockdown cells and JunB‐knockdown cells. (H) SRC‐1 cooperated with P65 to increase the promoter activities of MMP‐9 in 293 T cells. (I) SRC‐1 cooperated with c‐Jun to increase the promoter activity of MMP‐9 in 293 T cells. *p < 0.05, **p < 0.01, ***p < 0.001, ****p < 0.0001.

To investigate the regulatory effect of SRC‐1 on MMP‐9 promoter activity, a Luciferase assay was conducted using the MMP‐9 promoter reporter, spanning from −670 bp to +54 bp relative to the transcriptional starting site. As shown in Figure 3A, TPA treatment can increase the MMP‐9 promoter activity in HepG2 cells. Knockdown of SRC‐1 observably reduced the MMP‐9 promoter activity both in TPA treated and untreated group, suggesting that SRC‐1 can regulate MMP‐9 promoter activity.

SRC‐1 has been reported to enhance MMP‐9 transcription through coactivating PEA3^17^ and Ets2.^18^ To test the possibilities, Luciferase assay was conducted in 293 T cells. The results showed co‐overexpression of SRC‐1 and PEA3 significantly increased MMP‐9 promoter activity (Figure S2A). And co‐overexpression of SRC‐1 and Ets2 significantly increased MMP‐9 promoter activity (Figure S2B). As co‐overexpression of SRC‐1, PEA3 and Ets2 did not dramatically induce MMP‐9 promoter activity, suggesting that SRC‐1 may regulate MMP‐9 expression through coactivating other transcriptional factors in addition to PEA3 and Ets2.

NF‐κB and AP1 are main transcriptional factors of MMP‐9.^28^ To test whether SRC‐1 knockdown affected transcriptional activity of NF‐κB and AP‐1, NF‐κB and AP‐1 reporter assay were performed. SRC‐1‐knockdown significantly inhibited TPA‐induced NF‐κB and AP‐1 transcriptional activity (Figure 3B,C). And co‐overexpression of SRC‐1 and p65 or c‐Jun synergistically increased the NF‐κB and AP‐1 transcriptional activity (Figure S2C,D). These results suggested that SRC‐1 functions as a coactivator for NF‐κB and AP‐1 in HCC cells.

To confirm that NF‐κB and AP‐1 can regulate MMP‐9 promoter activity, we transiently knockdown NF‐κB and AP‐1 expression via transfecting siRNAs that are specific for p65 and JunB into HepG2 cells and then detected MMP‐9 mRNA levels and promoter activity. As shown in Figure 3D,E, knockdown of NF‐κB and JunB decreased the MMP‐9 promoter activity both in TPA treated and untreated group. Consistently, the mRNA levels of MMP‐9 were significantly decreased after knockdown of NF‐κB (Figure 3F) and AP‐1 (Figure 3G) when treated with TPA.

To validate the role of SRC‐1 as a coactivator for NF‐κB in enhancing MMP‐9 promoter activity, SRC‐1 and NF‐κB were overexpressed in 293 T and MHCC97H cells, and the activity of the MMP‐9 promoter was subsequently measured. Synergistically, co‐overexpression of SRC‐1 and NF‐κB significantly induced the enhance of MMP‐9 promoter activity, indicating that SRC‐1 coactivated NF‐κB to enhance MMP‐9 promoter activity (Figure 3H and Figure S3A). To further confirm that SRC‐1 worked as a coactivator for AP‐1 to enhance MMP‐9 promoter, SRC‐1 and AP‐1 were overexpressed in 293 T and MHCC97h cells, and MMP‐9 promoter activity was measured. The results showed co‐overexpression of SRC‐1 and AP‐1 induced significant increase of MMP‐9 promoter activity in 293 T and MHCC97h cells, indicating that SRC‐1 coactivated AP‐1 to enhance MMP‐9 promoter activity (Figure 3I; Figure S3B). These results suggested that SRC‐1 regulated MMP‐9 promoter activity through coactivating NF‐κB and AP‐1.

MMP‐9 gene expression is mainly regulated at the transcriptional level.^29^ The MMP‐9 promoter contains multiple cis‐elements that allow the regulation of MMP gene expression through a variety of trans‐activators including PEA3, Ets2, NF‐κB and AP‐1. We summarized the binding motifs of PEA3, Ets2 and NF‐κB and AP‐1 in the core MMP‐9 promoter region (−670 bp ~ +54 bp) according to the online tools JASPAR^30^ (http://jaspar.genereg.net/) and PROMO^31^, ^32^ (http://alggen.lsi.upc.es/cgi‐bin/promo_v3/promo/promoinit.cgi?dirDB=TF_8.3), as well as previous publications.^29^, ^33^ At least one PEA3, Ets2, NF‐kB and AP‐1 binding motifs were identified in the core promoter region of MMP‐9 (Figure S2E). The above studies can confirm that SRC‐1 coactivates NF‐κB and AP‐1 and simultaneously enhances MMP‐9 promoter activity. Further, we investigated the effect of SRC‐1 on the enrichment of NF‐κB and AP‐1 at the MMP9 promoter. As shown in Figure S3C,D, SRC‐1‐knockdown decreased the recruitment of NF‐κB and AP‐1 at the MMP9 promoter both in TPA treated and untreated group, indicating that SRC‐1 enhance MMP‐9 expression through enhancing recruitment of NF‐κB and AP‐1 to the promoter of MMP‐9.

### 3.4 SRC‐1 downregulation decreased cell invasion in HCC cells in vivo and predict a better survival

**FIGURE 4:**
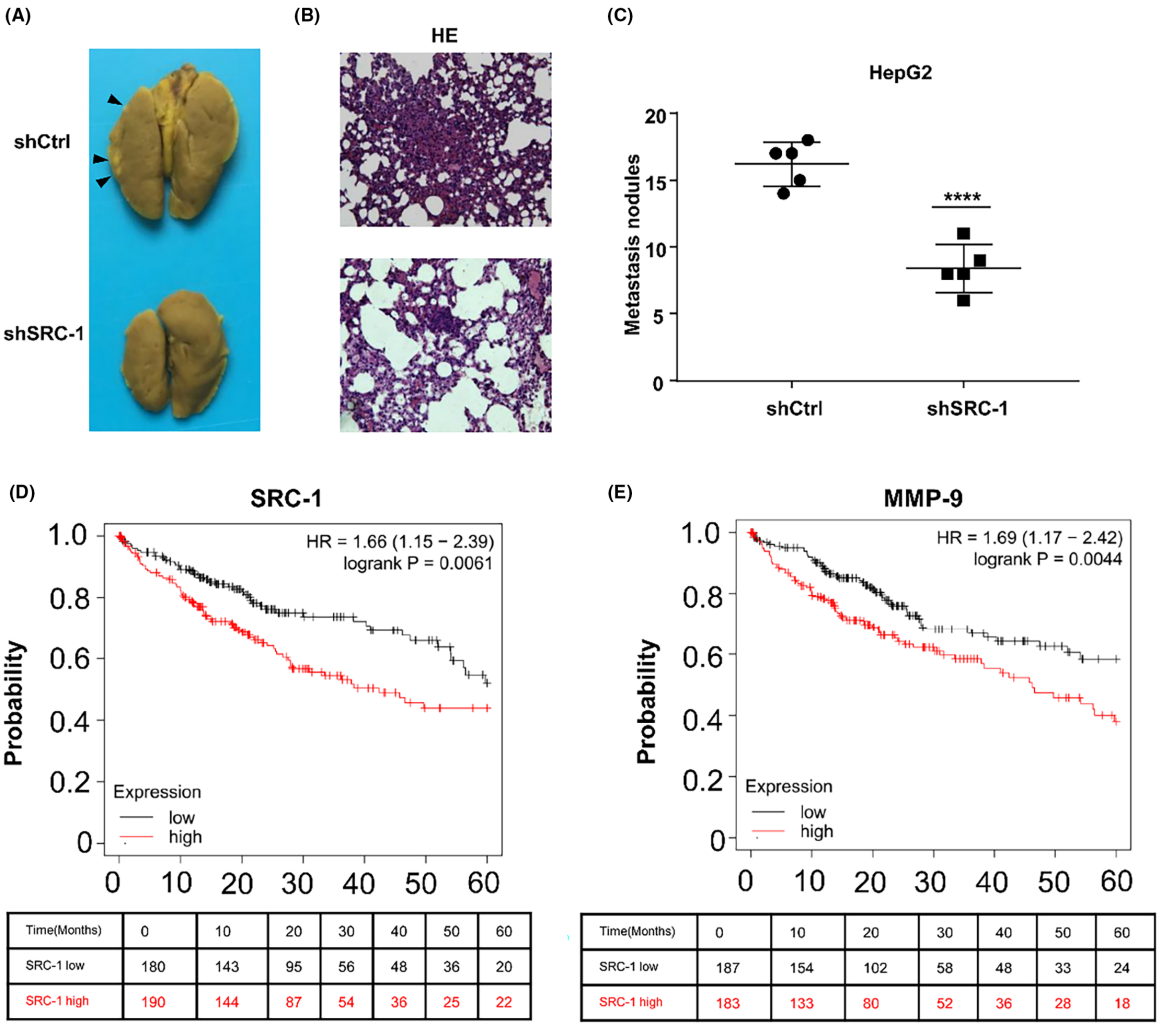
Downregulation of SRC‐1 decreased cell invasion in HCC cells in vivo. (A–C) Knockdown of SRC‐1 decreased lung metas‐tasis of HepG2 cells. The tumour number on the lung surface was counted. (D, E) SRC‐1 lower HCC patients have a better 5‐year survival than SRC‐1 higher HCC patients and MMP‐9 lower HCC patients have a better 5‐year survival than MMP‐9 higher HCC patents in the database KM‐plotter. ****p < 0.0001.

Furthermore, to investigate the potential role of SRC‐1 in HCC cell invasion in vivo, tumour metastasis was induced in BALB/c mice through tail vein injection of SRC‐1‐knockdown or control HepG2 cells. After 21 days, lungs were extracted for analysis. In the SRC‐1‐knockdown group, both the number and size of tumours on the lung surface were significantly reduced compared to the control group. (Figures 4A–C).

A considerable reduction in metastasis sites was observed in the SRC‐1 knockdown group (Figure 4A). Notably, the control group exhibited large tumour nodules, while only small tumour nodules were observed in the SRC‐1 knockdown group. (Figure 4B). The number of metastasis nodules was markedly reduced in SRC‐1‐knockdown group (Figure 4C). These results suggested that SRC‐1 knockdown decreased HCC cell invasiveness in vivo. To elucidate the expression of SRC‐1 in primary HCC tumours and circulating tumour cells (CTC), we analysed the GEO dataset GSE117623 using the online tool ctcRbase^34^ (http://www.origin‐gene.cn/database/ctcRbase/index.html). As depicted in Figure S4A, SRC‐1 expression was markedly elevated in CTC compared to primary tumours. This finding implies that HCC cells expressing higher levels of SRC‐1 possess an increased capability to invade the blood circulation system. Furthermore, in CTC, MMP‐9 expression was also significantly higher than that in primary tumours (Figure S4B), indicating that HCC cells with elevated MMP‐9 levels demonstrate an enhanced ability to invade the blood circulation system.

To figure out the expression of SRC‐1 in HCC primary tumour and HCC metastasis tumour, human HCC specimens were analysed in GEO database GSE40367. In a cohort of HCC patients, whether presenting lung metastasis or not, SRC‐1 expression (Figure S4C) and MMP‐9 expression (Figure S4D) were found to be higher in tumours with lung metastasis compared to primary HCC tumours. Similarly, in a cohort of HCC patients with or without adrenal gland metastasis, SRC‐1 expression (Figure S4E) and MMP‐9 expression (Figure S4F) exhibited higher levels in tumours with adrenal gland metastasis than in primary HCC tumours. Despite the limited sample size in this human HCC cohort, statistical significance was not achieved in these comparisons. However, a discernible trend was observed, suggesting that, in comparison to primary tumours, SRC‐1 and MMP‐9 expression tends to be increased in metastatic tumours. These findings imply that HCC tumours with elevated levels of SRC‐1 and MMP‐9 may have a heightened propensity to metastasize to other organs.

To examine the correlation between SRC‐1 expression and the 5‐year survival rate of HCC patients, we utilized the KM‐plotter database^35^ (http://kmplot.com/analysis/index.php?p=background), which provides survival data for various cancers, including HCC. As presented in Figure 4D, HCC patients with lower SRC‐1 expression exhibited a superior 5‐year survival compared to those with higher SRC‐1 expression. Similarly, for MMP‐9, patients with lower expression demonstrated a better 5‐year survival rate than those with higher MMP‐9 expression (Figure 4E). These findings suggest that the downregulation of SRC‐1 is associated with a more favourable survival outcome.

### 3.5 Bufalin treatment inhibited HCC cell invasiveness

**FIGURE 5:**
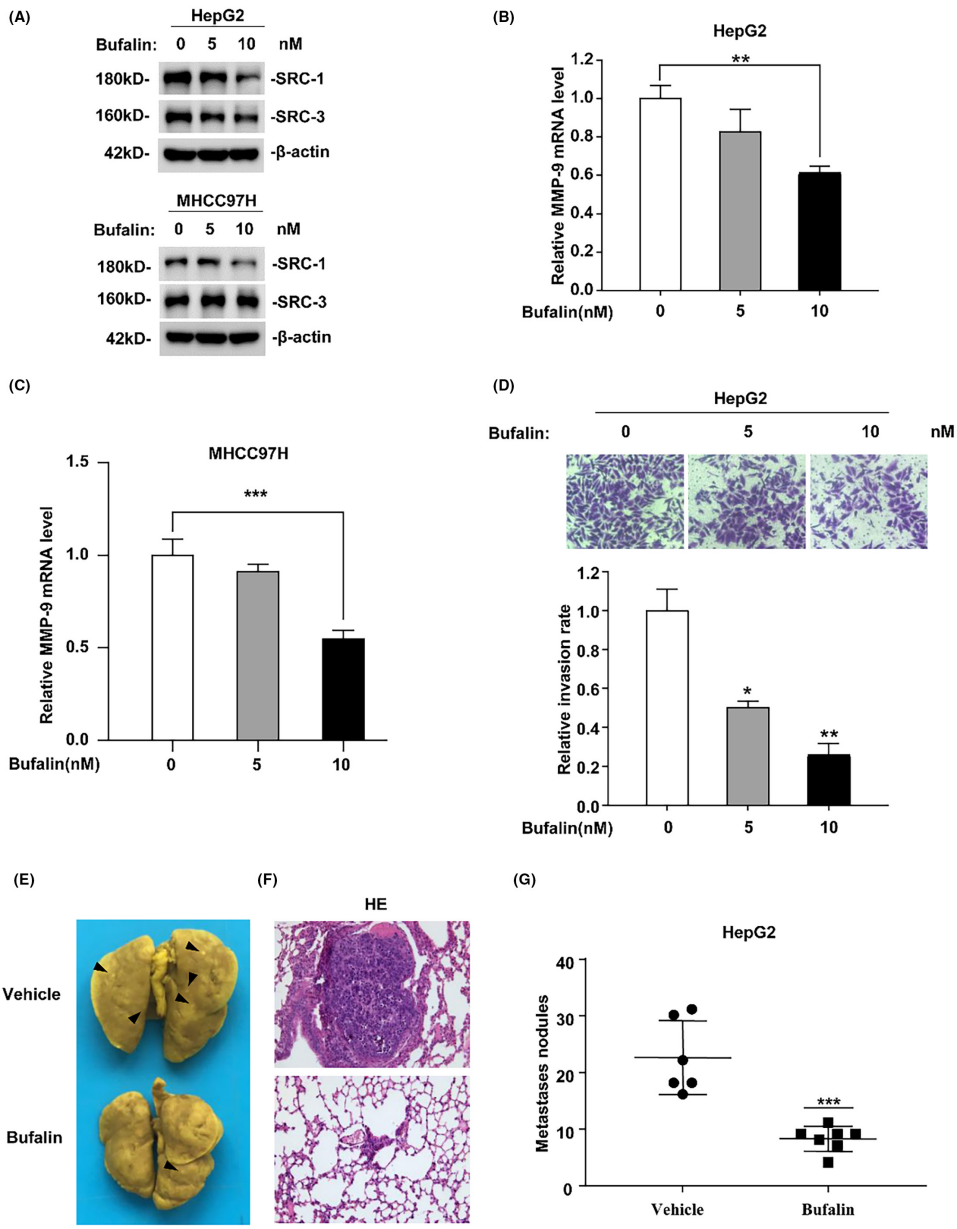
Bufalin treatment inhibited HCC cell invasiveness. (A) Effect of Bufalin on SRC‐1 and SRC‐3 protein level in HepG2 and MHCC97H cells. (B, C) MMP‐9 mRNA levels were significantly decreased after bufalin treatment both in HepG2 and MHCC97H cells. (D) Bufalin treatment obviously decreased the invasive ability of HepG2. The calculated number from triplicate was shows Images and quantitation cells penetrating through the matrigel‐coated membrane. (E–G) Bufalin treatment inhibited lung metastasis of HepG2 cells. The tumour number on the lung surface was calculated. *p < 0.05, **p < 0.01, ***p < 0.001.

Given that SRC‐1 downregulation led to a decrease in HCC cell invasiveness both in vitro and in vivo, inhibiting SRC‐1 emerges as a potentially effective strategy for controlling HCC metastasis. To assess the impact of SRC‐1 inhibition on HCC cell invasiveness, we selected Bufalin, a well‐known SRC‐1 inhibitor, for treatment in both HCC cells and metastasis tumour mouse models. The reduction of SRC‐1 protein levels by Bufalin in HepG2 and MHCC97H cells was validated through Western blot analysis (Figure 5A). The results indicated a significant decrease in MMP‐9 mRNA levels following SRC‐1 inhibitor treatment in both HepG2 (Figure 5B) and MHCC97H cells (Figure 5C). Moreover, Bufalin treatment led to a substantial decrease in cell invasiveness in HepG2 cells (Figure 5D), suggesting that the SRC‐1 inhibitor significantly attenuated HCC invasion in vitro.

To assess the impact of the SRC‐1 inhibitor on HCC cell metastasis in vivo, HepG2 cells were intravenously injected into nude mice and treated with Bufalin or vehicle. Metastasis sites were observed in the lungs of vehicle‐treated mice (Figure 5E). In contrast, a notably reduced number of metastasis sites were observed in the lungs of Bufalin‐treated mice. Large tumour nodules were evident in the vehicle‐treated group, whereas only small tumour nodules were found in the Bufalin‐treated group (Figure 5F). Furthermore, the total number of metastasis nodules was significantly decreased in the Bufalin‐treated group (Figure 5G). Considering that bufalin's molecular targets also include SRC‐3, which can increase MMP‐9 activity to enhance HCC cells migration and invasion, we examined the expression of SRC‐3 after bufalin treatment in HepG2 and MHCC97h cells. The results revealed a significant reduction in SRC‐3 expression in HepG2 cells upon bufalin treatment (Figure 5A). However, there was no apparent impact on SRC‐3 protein levels in MHCC97h cells (Figure 5A). Furthermore, under bufalin treatment, we conducted a rescue experiment by SRC‐3 overexpression in HepG2 cells. The results indicated that overexpression of SRC‐3 only partially restored the expression of MMP‐9 (Figure S5A, B). These results suggest that SRC‐1 indeed plays a regulatory role in the expression of MMP‐9 and cell metastasis, and SRC‐1 inhibitor treatment significantly decreased HCC cell metastasis in vivo.

### 3.6 Depletion of SRC‐1 decreases MEF cells invasion

**FIGURE 6:**
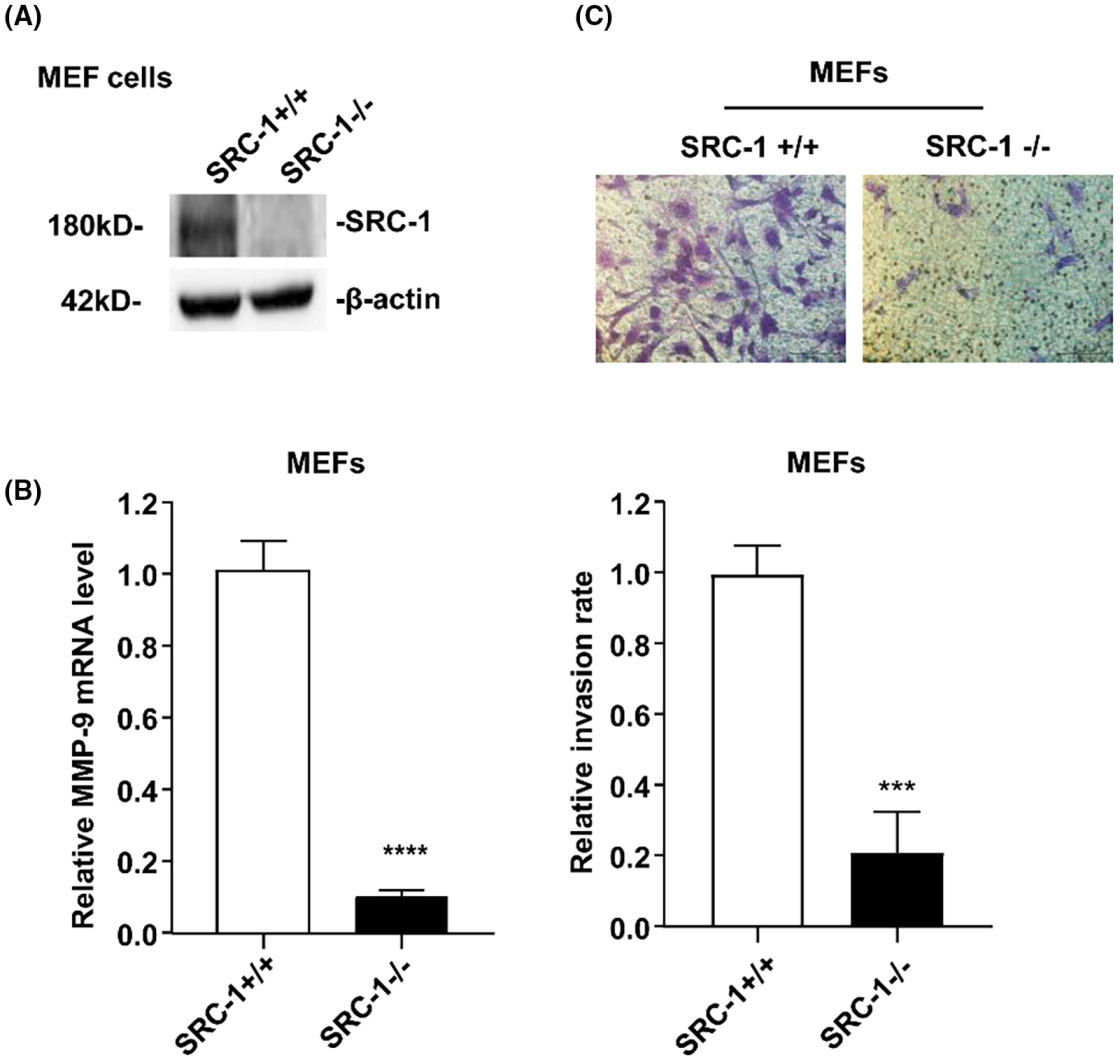
(A) SRC‐1 knockout in MEF cells was confirmed by Western blot analysis. (B) SRC‐1 knockout in MEF cells markedly re‐duced MMP‐9 mRNA level. (C) Downregulation of SRC‐1 decreased the invasive ability of MEF cells. Images and quantitation of SRC‐1 transient knockdown and control cells penetrating through the matrigel‐coated membrane. ***p < 0.001, ****p < 0.0001.

To ascertain whether the effect of SRC‐1 on cell invasiveness was specific to HCC cells, we isolated MEF cells from SRC‐1 wild‐type and SRC‐1 knockout mice. SRC‐1 knockout was validated by Western blot in MEF cells (Figure 6A). Similarly, SRC‐1 knockout in MEF cells significantly reduced MMP‐9 mRNA levels (Figure 6B). Notably, the number of cells invading through Matrigel‐embedded transwell assays was substantially reduced in SRC‐1 knockout MEF cells (Figure 6C). These results suggest that SRC‐1 regulates cell invasiveness extends beyond HCC cells and can be effective in other tissues as well.

## 4 DISCUSSION

HCC, constituting 75%–85% of liver cancers, ranks as the sixth most commonly diagnosed cancer and the fourth leading cause of cancer‐related mortality globally. It stands as the second most frequent cause of cancer death in men, surpassed only by lung cancer.^1^ HCC is characterized by a challenging prognosis, rapid growth, vascular invasion, metastasis, and recurrence. Current treatment strategies for metastatic liver cancer, involving radiation therapy and chemotherapy, often lead to significant side effects, considerable pain for patients, and may not achieve complete eradication of all cancer cells. Consequently, there is a critical need to unravel the molecular mechanisms driving HCC metastasis and develop effective therapies to control this process. Despite advances in understanding the molecular basis of metastasis, there remains a substantial gap in our knowledge, and effective therapeutic interventions for metastasis control are still elusive.

In the metastasis of liver cancer, the lungs, brain and bones are commonly affected sites. Various factors, including miRNA, transcriptional factors and transcriptional coactivators mediate the metastasis of HCC. Among these, the SRC family, particularly SRC‐1 and SRC‐3, exhibit elevated expression in numerous cancers, contributing to cancer progression and metastasis. Clinical observations have linked increased expression of SRC‐3 with regional invasion and lymph node metastasis.^36^ Various cancers induced by aberrant SRC‐3 overexpression were invasive and metastatic in a SRC‐3‐transgenic mouse model.^37^, ^38^ Inversely, SRC‐3‐knockout mouse model presented inhibited cancer invasion and metastasis.^39^, ^40^, ^41^ Knockout of SRC‐1 in mouse mammary tumour virus–polyoma middle T (PyMT) impeded metastasis without impacting primary tumour formation.^13^ SRC‐1 upregulates the expression of ERBB2, colony‐stimulating factor 1 and TWIST1 thereby promoting metastasis in breast cancer.^13^, ^42^ Previously, we found that SRC‐1 and SRC‐3 were both higher expression in HCC specimens.^15^, ^24^ SRC‐3 accelerated HCC progression via enhancing cell proliferation and invasion.^24^, ^25^ SRC‐3 promotes HCC cell invasiveness via coactivating HBX and NF‐κB/AP‐1 to enhance MMP‐9.^25^ SRC‐1 exhibited upregulation in 60% of 40 HCC specimens, contributing to the progression of HCC by enhancing Wnt/β‐Catenin signalling.^15^ Specifically, we utilized publicly available data (GSE212943) to analyse the epigenetic characteristics of SRC1 in normal hepatocytes (L‐02) and HCC cells (PLC). Our findings reveal enhanced modification of active histones, particularly H3K27ac, in the promoter region of SRC1 in HCC cells (PLC), suggesting a heightened activation of SRC1 expression (Figure S5C). Furthermore, we identified the presence of enhancers marked by H3K27ac and Loop/TAD boundaries marked by CTCF and SMC3 in the SRC1 gene region (Figure S5D). According to the ring extrusion model,^43^ in normal liver cells (L‐02), enhancers on SRC1 interact with distal promoters, and chromatin structural proteins CTCF and SMC3 form a chromatin ring. In this scenario, RNA polymerase is impeded from crossing the boundary of the ring during SRC1 transcription, leading to transcriptional suppression. Contrastingly, in HCC cells (PLC), the chromatin loop between enhancers and distal promoters on SRC1 disappears, enabling RNA polymerase to transcribe SRC1 normally (Figure S5E). These observations shed light on the dynamic chromatin interactions and structural changes associated with SRC1 regulation, providing insights into the differential transcriptional regulation of SRC1 in normal and HCC.

The role of SRC‐1 in HCC metastasis has remained elusive. In the current study, SRC‐1 knockdown demonstrated a reduction in HCC cell metastasis both in vitro and in vivo. Clinically, SRC‐1 exhibited higher expression in CTCs than in primary tumours. Elevated SRC‐1 expression was observed in lung and adrenal gland metastasis tumours compared to primary HCC. These findings suggest a pivotal role for SRC‐1 in HCC metastasis, with higher SRC‐1 expression correlating with poorer overall survival in HCC patients. Treatment with the SRC‐1 inhibitor Bufalin significantly inhibited HCC cell invasiveness in both in vitro and in vivo settings. These results highlight the potential of SRC‐1 inhibition as a novel therapeutic candidate for HCC treatment.

MMP‐9, implicated in extracellular matrix breakdown, plays a pivotal role in tumour invasion, metastasis, angiogenesis and the regulation of the tumour microenvironment.^16^ This is corroborated by the findings indicating elevated MMP‐9 expression in circulating tumour cells, lung and adrenal gland metastasis tumours, and its correlation with a worse overall survival rate. In the present study, SRC‐1 knockdown significantly reduced the mRNA, enzymatic activity, and promoter activity of MMP9 in HCC cells, both with and without TPA treatment. This suggests that SRC‐1 may drive HCC metastasis by enhancing MMP‐9 expression. The MMP‐9 promoter contains cis‐elements allowing for regulation by PEA3, Ets2, NF‐κB and AP‐1. SRC‐1 directly binds to PEA3 and the VEGF and MMP‐9 promoters under VEGF and VEGF inducer GS4012 treatment in glioma cells.^17^ SRC‐1 and Ets2 interact to regulate expression of MMP9 target genes in Aromatase inhibitors resistance in breast cancer cells.^18^ However, co‐overexpression of SRC‐1 and PEA3 increased MMP‐9 promoter activity by 3.9‐fold, less than the additive effect of SRC‐1 (2‐fold) and PEA3 (2.8‐fold) alone. Co‐overexpression of SRC‐1 and Ets2 increased MMP‐9 promoter activity by 5.6‐fold, equals the additive effect of SRC‐1 (2‐fold) and PEA3 (3.6‐fold) alone. As co‐overexpression of SRC‐1 and PEA3/Ets2 did not dramatically induce MMP‐9 promoter activity, it is suggested that SRC‐1 regulates MMP‐9 expression through coactivating other transcriptional factors in addition to PEA3 and Ets2. NF‐κB and AP‐1 signalling are required for MMP‐9 transcription when treated with TPA.^28^

As SRC‐1 is known to coactivate NF‐κB^9^ and AP‐1,^10^ and SRC‐3 has been reported to enhance MMP‐9 promoter activity through coactivating NF‐κB and AP‐1,^24^ we hypothesize that SRC‐1 regulates MMP‐9 expression by coactivating NF‐κB and AP‐1. Transient knockdown of SRC‐1 significantly inhibited TPA‐induced NF‐κB transcriptional activity. Co‐overexpression of SRC‐1 and P65 synergistically increased NF‐κB transactivation activity, indicating SRC‐1 function as a coactivator for NF‐κB in HCC cells. TPA significantly increased AP‐1 transcriptional activity in HepG2 cells, and co‐overexpression of SRC‐1 and c‐JUN synergistically enhanced AP‐1 transcriptional activity. These results suggest that SRC‐1 functions as a coactivator for AP‐1 in HCC cells. Co‐overexpression of SRC‐1 and NF‐κB synergistically induced MMP‐9 promoter activity, indicating that SRC‐1 coactivated NF‐κB to enhance MMP‐9 promoter activity. Co‐overexpression of SRC‐1 and AP‐1 synergistically induced MMP‐9 promoter activity, indicating that SRC‐1 coactivated AP‐1 to enhance MMP‐9 promoter activity. These results suggested that SRC‐1 regulated MMP‐9 promoter activity through coactivating NF‐κB and AP‐1. Futher ChIP assays results disclosed that SRC‐1 enhance the recruitment of NF‐kB and AP‐1 to the promoter of MMP‐9.

In view of that SRC‐1 and SRC‐3 act crucial characters in HCC metastasis and are simultaneously highly expressed in HCC patients, we wonder whether SRC‐1 and SRC‐3 would work together to enhance MMP‐9 expression. According to the protein levels of SRC‐1 and SRC‐3, HCC specimens were divided into 4 groups: SRC‐1(−)/SRC‐3 (−), SRC‐1 (−)/SRC‐3(+) and SRC‐1(+)/SRC‐3(−), and SRC‐1(+)/SRC‐3(+) HCC specimens.^15^ Due to the limited number of HCC specimens in each group, there was no statistical difference between these four groups. However, we can still observe a trend that MMP‐9 mRNA level was higher in SRC‐1 (−)/SRC‐3(+) and SRC‐1(+)/SRC‐3(−), and SRC‐1(+)/SRC‐3(+) groups than that inSRC‐1(−)/SRC‐3 (−), with SRC‐1(+)/SRC‐3(+) group exhibited the highest MMP‐9 mRNA levels (Figure S6A). Whether SRC‐1 and SRC‐3 would work together to enhance MMP‐9 expression needs further study to validate it.

Taken together, the model for the role of SRC‐1 in HCC metastasis can be postulated in Figure S6B. With the stimulus such as TPA, SRC‐1 is recruited to the promoter of MMP‐9 through PEA3, Ets2, NF‐κB and AP‐1. MMP‐9 expression is then enhanced and promotes HCC metastasis. When treated with SRC‐1 inhibitor Bufalin, SRC‐1 protein levels are decreased. Less SRC‐1 is recruited to the promoter of MMP‐9 and MMP‐9 expression is subsequently decreased. This will result in the decreased HCC metastasis.

## 5 CONCLUSIONS

In conclusion, this study demonstrates that SRC‐1 promotes HCC metastasis via enhancing MMP‐9 expression, indicating that inhibition of SRC‐1 may be an efficient therapeutic strategy for controlling HCC metastasis.

## AUTHOR CONTRIBUTIONS

**Zhangwei Tong:** Investigation (lead); methodology (equal); writing – original draft (equal). **Yong Zhang:** Data curation (equal); investigation (equal); methodology (equal); writing – original draft (equal). **Peng Guo:** Formal analysis (equal); investigation (equal); methodology (equal). **Wei Wang:** Data curation (equal); formal analysis (equal). **Qiang Chen:** Data curation (equal); software (equal). **Jing Jin:** Data curation (equal); formal analysis (equal). **Shixiao Liu:** Data curation (equal); formal analysis (equal). **Chundong Yu:** Supervision (equal); writing – review and editing (equal). **Pingli Mo:** Conceptualization (equal); project administration (equal); writing – review and editing (equal). **Lei Zhang:** Funding acquisition (equal); supervision (equal); validation (equal); writing – review and editing (equal). **Junli Huang:** Funding acquisition (equal); project administration (equal); resources (equal); writing – review and editing (equal).

## FUNDING INFORMATION

This research was funded by the National Natural Science Foundation of China (no. 82073090), Shanxi Province ‘136’ Revitalization Medical Project Construction Funds, Guidance project on the medical health of Xiamen (3502Z20214ZD1191), Xiamen Municipal Bureau of Science and Technology.

## CONFLICT OF INTEREST STATEMENT

The authors declare there is no conflict of interests.

## CONSENT

Patients signed informed consent regarding publishing their data and photographs.

## Supporting information


Figure S1.
Figure S2.
Figure S3.
Figure S4.
Figure S5.
Figure S6.

## Data Availability

The datasets used or analysed during the current study are available from the corresponding author on reasonable request.
