# Supplementary material for: Steroid receptor coactivator 1 promotes human hepatocellular carcinoma invasiveness through enhancing MMP‐9
Source: J Cell Mol Med. 2024 Mar 20;28(7):e18171. doi: 10.1111/jcmm.18171 (PMC10951881; doi:10.1111/jcmm.18171)

Supplementary Figure 1


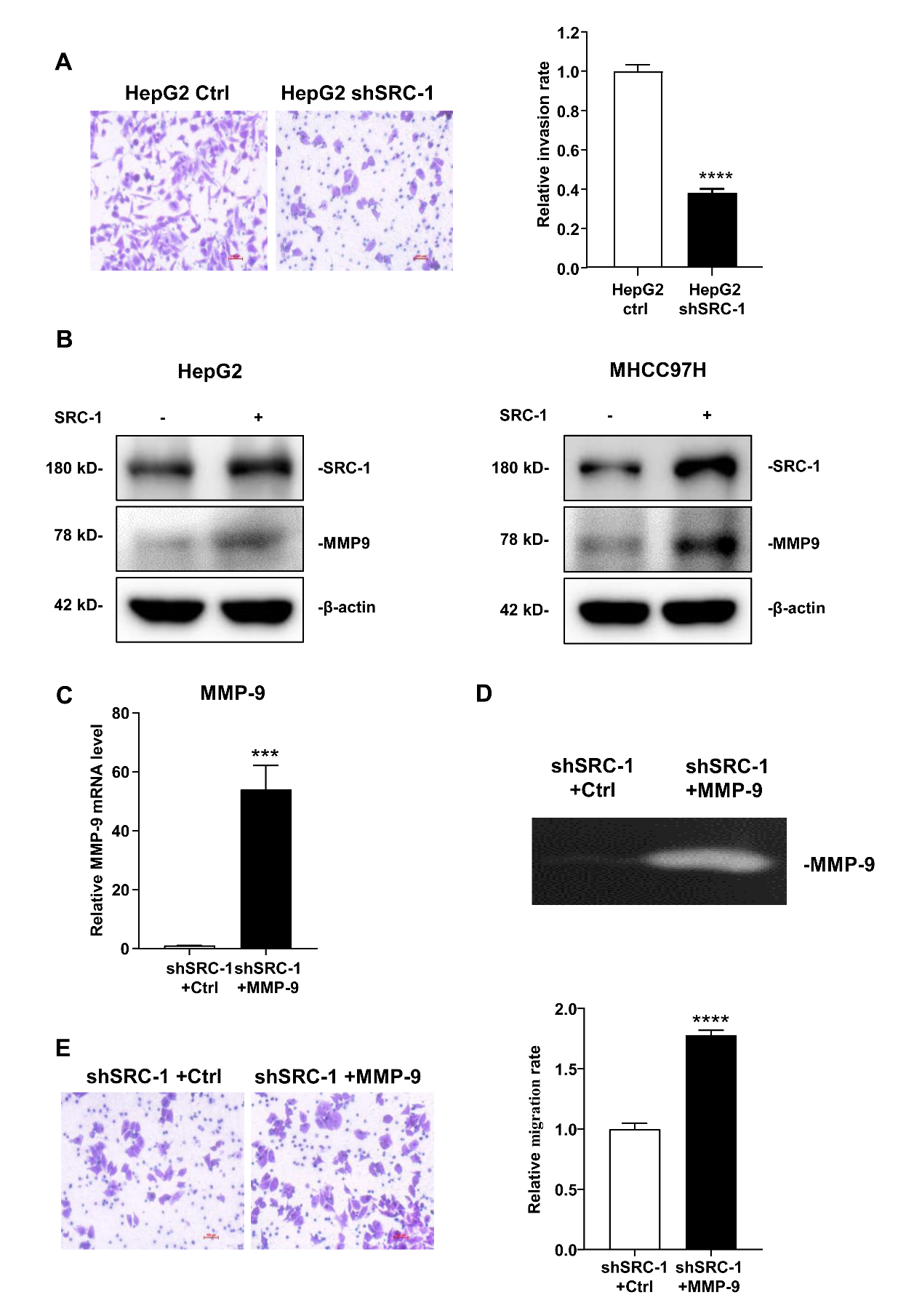


Supplementary Figure 2


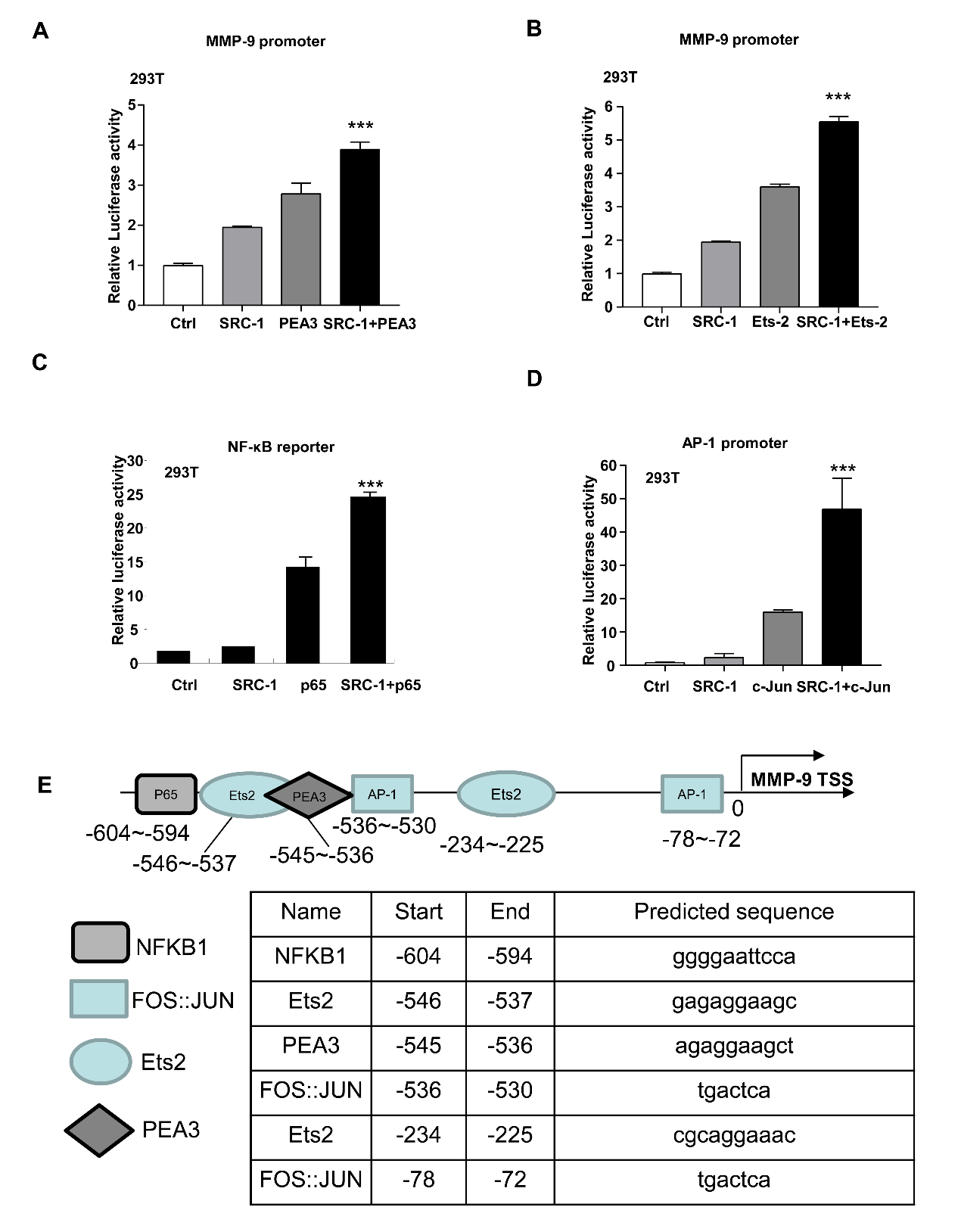


Supplementary Figure 3


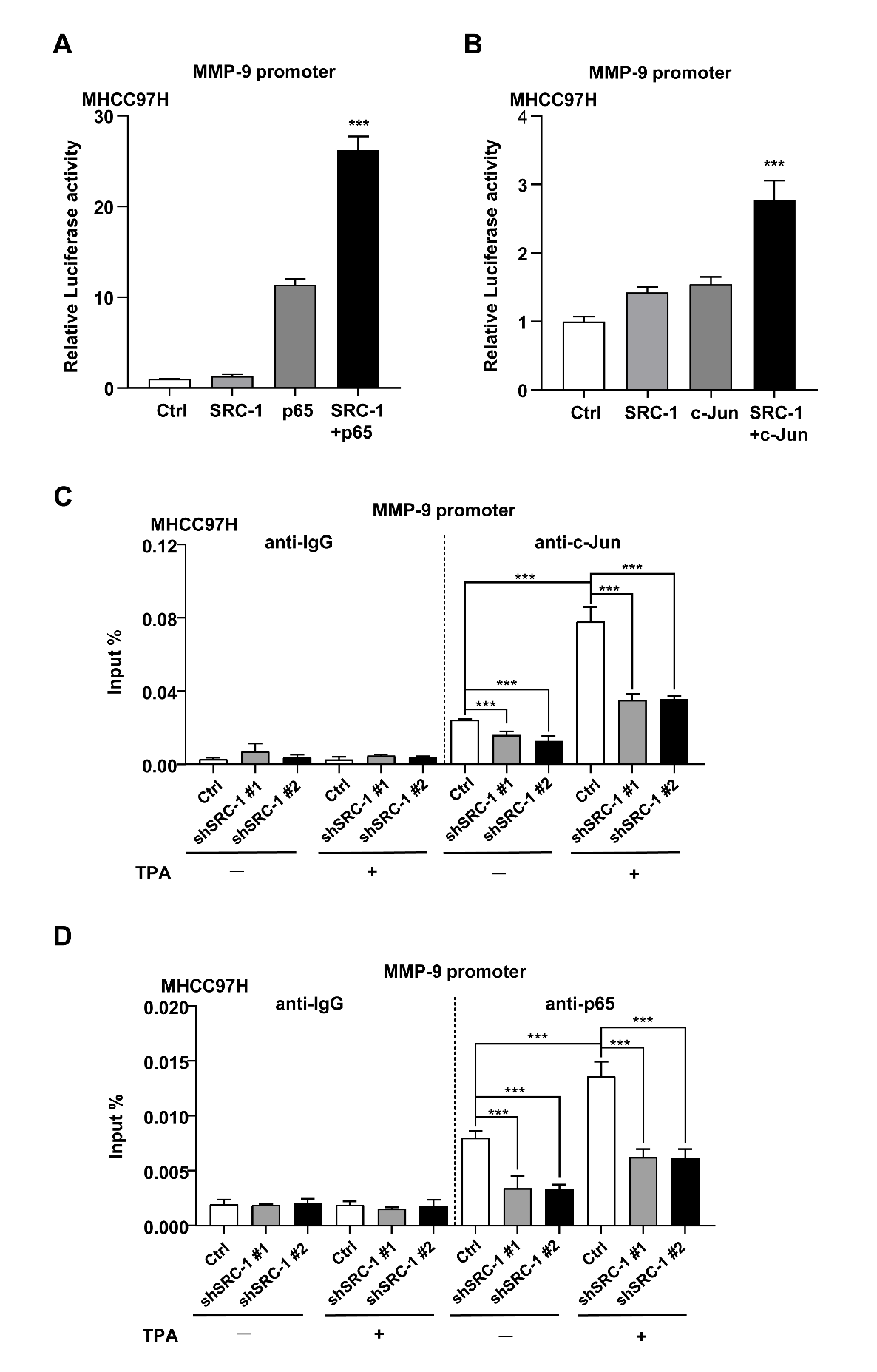


Supplementary Figure 4


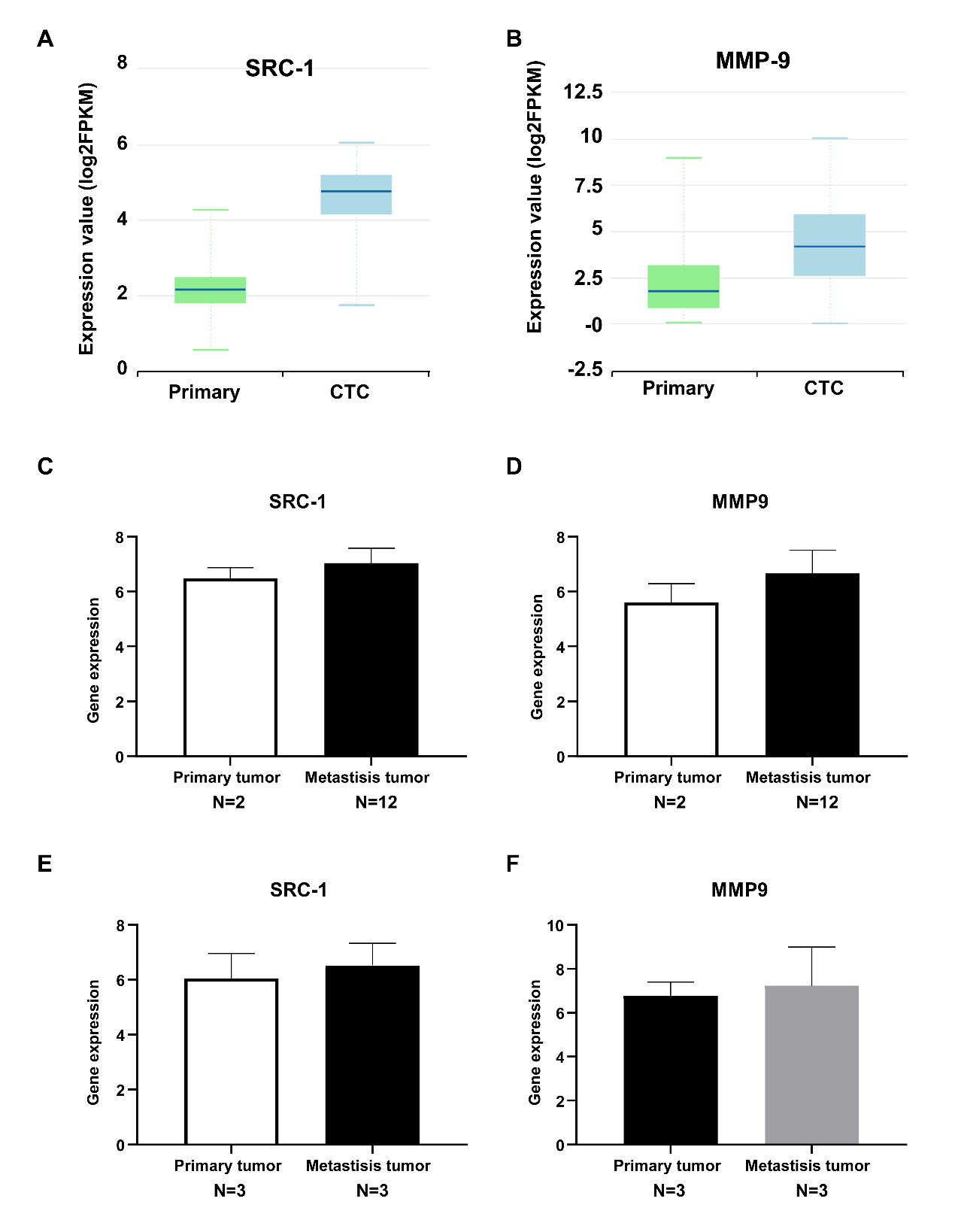


Supplementary Figure 5


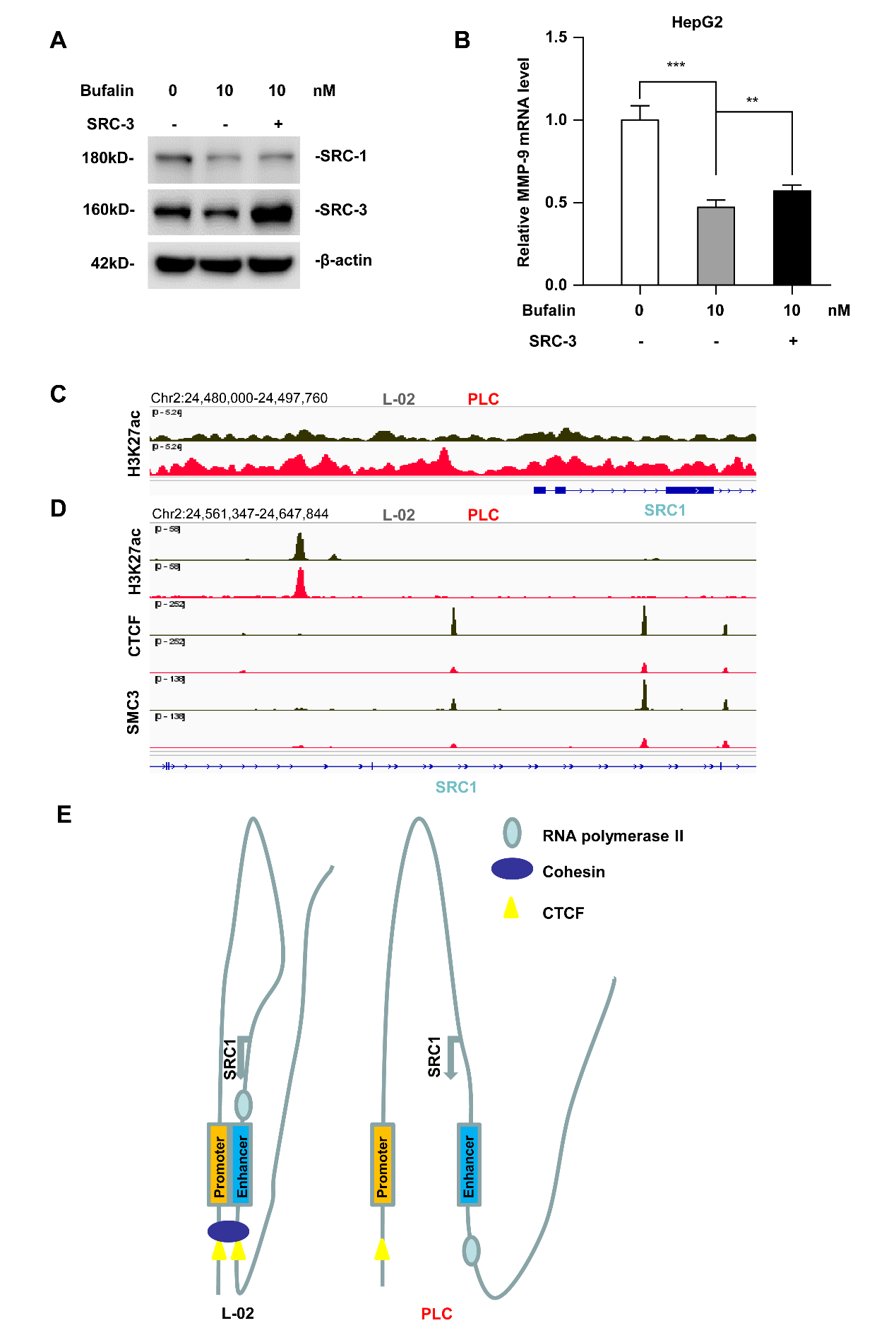


Supplementary Figure 6


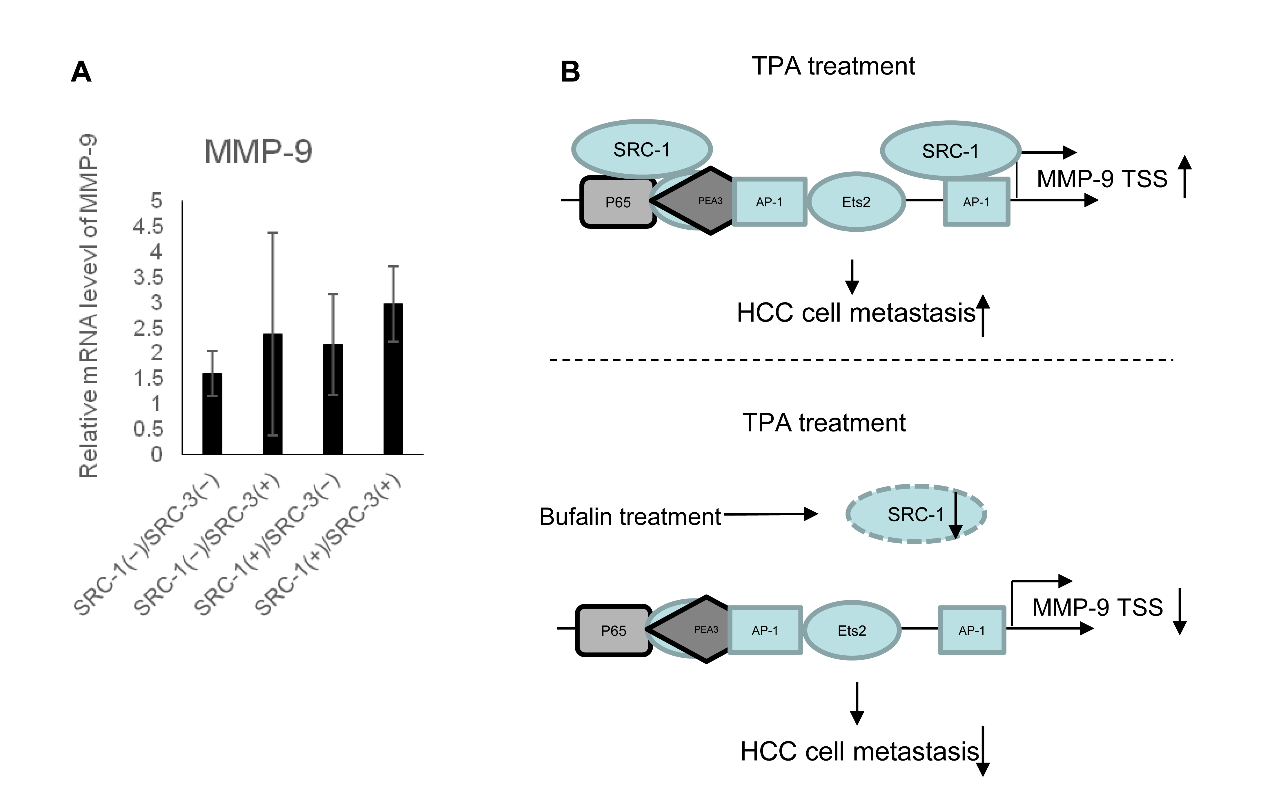

Supplement: Supplementary file 1 — Figure S1. Figure S2. Figure S3. Figure S4. Figure S5. Figure S6. [file JCMM-28-e18171-s001.docx]
